# Understanding of COVID-19 Pathology: Much More Attention to Plasma Proteins

**DOI:** 10.3389/fimmu.2021.656099

**Published:** 2021-03-24

**Authors:** Masahiro Nishibori, Barbara S. Stonestreet

**Affiliations:** ^1^ Department of Pharmacology, Okayama University Graduate School of Medicine, Dentistry and Pharmaceutical Sciences, Okayama, Japan; ^2^ Department of Pediatrics, Women & Infants Hospital of Rhode Island, The Alpert Medical School of Brown University, Providence, RI, United States

**Keywords:** COVID-19, histidine-rich glycoprotein, inter-alpha inhibitor proteins, NETs, ARDS, immunothrombosis

## Introduction

The recent outbreak of the novel coronavirus infection (COVID-19) has produced a world-wide health problem (1). COVID-19 is extremely infectious and has high mortality rate in vulnerable individuals (2). On the other hand, there could exist a large percentage of infected patients, which exhibit mild symptoms or even no apparent symptoms. This situation could make surveillance control difficult to prevent the spread of infection efficiently. In the case of severely ill patients, the rapid and progressive development of the acute respiratory response syndrome (ARDS) is critical and often fatal, and associated with organ failure including cardiovascular disorders, lung embolism, acute kidney injury and DIC (1).

## COVID-19 and Thrombosis/Embolism

Clinical features in patients with severe COVID-19 include increases in D-dimers and fibrin product degradation as well as elevations in von Willebrand factor and soluble P-selectin, suggesting the existence of endotheliopathy along with platelet activation in COVID-19-associated coagulopathy (2–4). These findings were consistent with the fact that obesity, diabetes, and hypertension are the risk factors and major comorbidities for severe COVID-19 (4). Angiotensin converting enzyme-2, a receptor for the spike protein of severe acute respiratory syndrome coronavirus-2 (SARS-Cov-2), is expressed on the surface of vascular endothelial cells as well as respiratory epithelial cells. Thus, vascular endothelial cells appear to be a target cell for SARS-Cov-2 infection and a primary site for invasion and replication that may induce abnormalities in endothelial cells, resulting in prothrombotic conditions in the vasculature. Findings from patient autopsies strongly suggest the presence of thrombus formation in lung vasculature as well as thromboembolism in the peripheral vessels (3–5). Thromboses of the coronary or carotid arteries have also been reported to result in sudden onset of fatal cardiovascular events (6, 7). Thus, in addition to ARDS, the wide range of thromboses and embolism could be a fundamental pathophysiological component of severe COVID-19 (5).

## COVID -19 and Neutrophil Extracellular Traps

Recent histopathological analyses of patients with COVID-19 have suggested that neutrophil extracellular traps (NETs) with web-like DNAs released from the cells bearing myeloperoxidase, neutrophil elastase and citrullinated histone 3 on DNA (8, 9), are involved in thrombus formation in the lung vasculature, suggesting a form of immunothrombosis (10, 11). NETs are thought to be induced by excessive activation of neutrophils resulting in an increase in cytosolic calcium and activation of protein kinase C, followed by activation of NADPH oxidase. The resulting production of reactive oxygen species is required for the translocation of neutrophil elastase and myeloperoxidase as well as citrullination of histone 3 and de-condensation of chromatin DNA. The immunothrombus starts from the adhesion of NETs on vascular endothelial cells, followed by platelet aggregation on the NETs along with fibrin deposition (12), suggesting a very crucial and important role for NETs in the development of immunothrombi.

The activation state of vascular endothelial cells is another factor that can predispose to the development of thrombosis (12). Endothelial cells expressing ACE-2, a receptor for the spike protein of SARS-Cov-2, can be activated after infection with SARS-Cov-2. Once the endothelial cells are activated, the interactions between endothelial cells and neutrophils, particularly the NETs, will be increased. The extracellular release of ROS and proteinases from neutrophils further facilitate the activation of endothelial cells, resulting in an increase in the expression of adhesion molecules on the surface of endothelial cells. Thus, dysregulated NETs on endothelial cells form a vicious cycle between them, followed by platelet aggregation on NETs. The coagulation cascade may initiate on the surface of aggregated platelets as well as on tissue factor-expressing endothelial cells. Finally, a series of events beginning with the NETs and the damaged vascular endothelial cells losing their anti-coagulant properties will result in immunothrombus formation (3, 4). Some groups have also reported the existence of NETs in interstitial and alveolar spaces beyond the vasculature in the lung parenchyma (13, 14), contributing to the development of ARDS. Therefore, the regulation of NETs has been suggested to be one of the directions for the treatment of COVID-19 (15, 16).

## Inhibition of Nets by Histidine-Rich Glycoprotein

Histidine-rich glycoprotein (HRG), a 75 kDa plasma protein primarily produced by the liver, has multiple functions. HRG regulates angiogenesis, coagulation/fibrinolysis, host defenses, dead cell clearance and tumor growth (17). Recently, our group reported that a rapid decrease in plasma HRG in patients with septic disorders could trigger multiple organ failure (MOF) because HRG maintains circulating neutrophils in a resting state and protects vascular endothelial cells from excessive activation by several types of stimulants, thereby preventing NETosis and immunothrombosis (18). The loss of such homeostasis because of reductions in HRG may predispose to a cascade of events in septic patients resulting in respiratory failure, circulatory shock, renal failure and DIC (18, 19). We have already demonstrated that HRG inhibits NETs *in vitro* (20) and *in vivo* (18) and reduces ROS production (21). In addition, HRG protects vascular endothelial cells from excessive activation, inhibits the expression of adhesion molecules, inhibits HMGB1 release and suppresses cytokine production (22, 23). All these effects of HRG on vascular endothelial cells inhibit the interactions between vascular endothelial cells and neutrophils/platelets, maintaining the anti-thrombotic condition at the interfaces of the circulating blood and vascular wall (23). Moreover, HRG suppresses the intrinsic pathway of the coagulation cascade directly by inhibiting XIIa (24). A clinical study demonstrated that the plasma levels of HRG were lower in non-survivors than in survivors on the admission day in ICU patients (19). Therefore, plasma HRG could be a superior biomarker of sepsis compared with the current standard of care, which uses procalcitonin and presepsin as indicators of sepsis (19, 25).

## Inter α-Inhibitor Proteins as Anti-Septic Plasma Proteins

**Figure 1:**
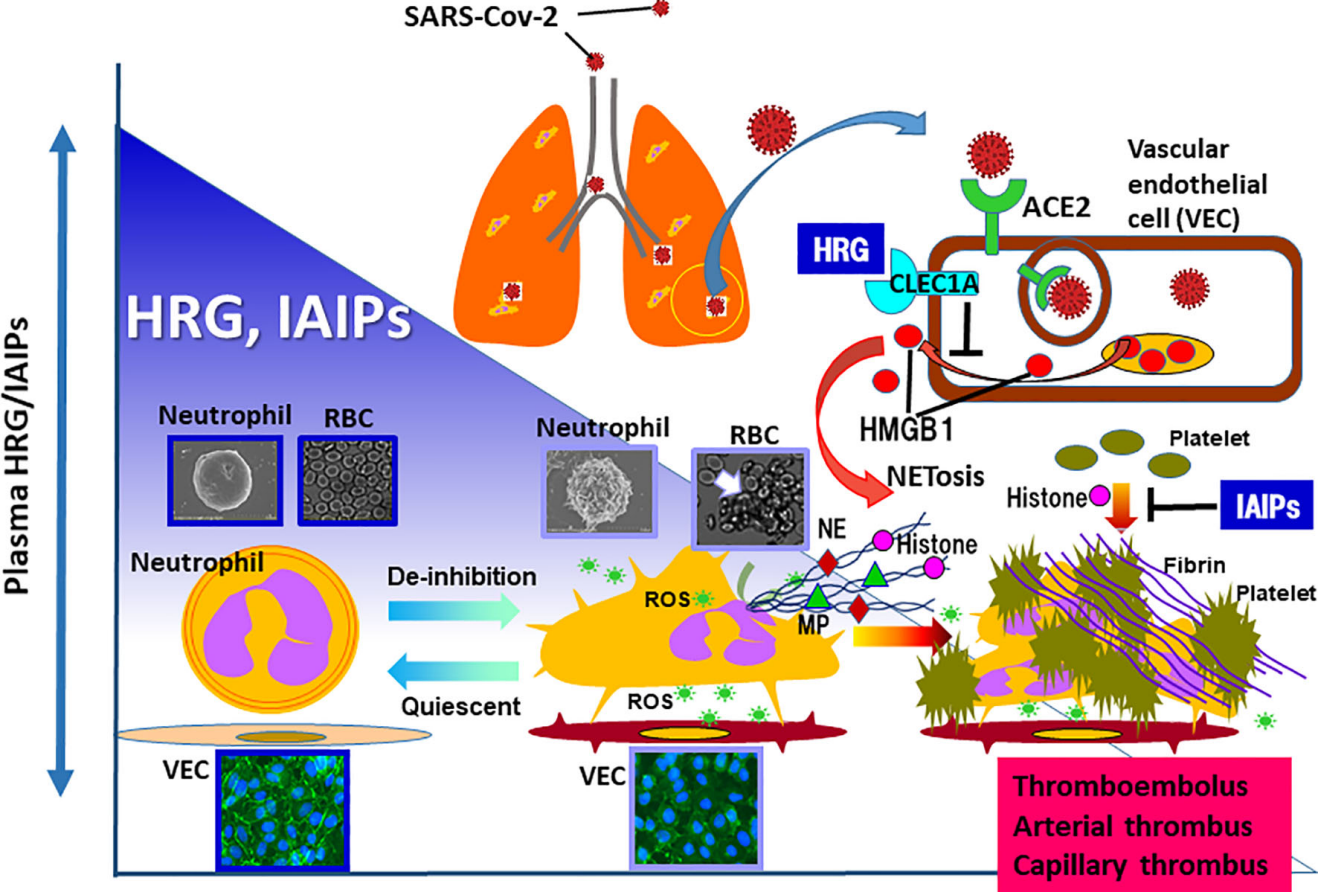
In the animal models of sepsis as well as septic patients, the remarkable decrease in plasma levels of HRG and IAIPs are evident. HRG controls the shape and function of neutrophils and inhibits NETosis in neutrophils. In addition, HRG protects vascular endothelial cells from excessive activation by inhibiting HMGB1 release from the cells, which was mediated by the stimulation of CLEC1A. IAIPs also inhibit histone-induced platelet aggregation and exert protective effects on vascular endothelial cells. Rapid decreases in plasma HRG and IAIPs will diminish these homeostatic effects, leading to immunothrombosis. Therefore, it is particularly important to ascertain changes in plasma levels of HRG and IAIPs in patients with COVID-19. ROS, reactive oxygen species; NE, neutrophil elastase; MP, myeloperoxidase.

Inter α-inhibitor proteins (IAIPs) are a family of structurally related serine proteinase inhibitors found in plasma in relatively high concentrations (around 500 μg/ml). The major forms of these proteins in human plasma consist of two heavy chains and a single light chain called bikunin, covalently linked through esterification of chondroitin sulfate chain on bikunin. The minor forms contain different kinds of a single heavy chain coupled to bikunin in the same manner (26). IAIPs play a role in the regulation of a variety of responses including inflammation, wound healing, ovulation and cancer invasion/metastasis (26). In a neonatal animal model of sepsis, IAIPs decreased significantly after the induction of sepsis and supplementary therapy improved the lethality (27, 28). In addition, IAIPs have been suggested as both diagnostic and therapeutic agents in neonatal and adult septic patients (29, 30). IAIPs inhibit platelet aggregation induced by histone H3 (31) and suppress the spontaneous ROS production in neutrophils (32). Moreover, phenotypic analysis of IAIP knockout mice and the relationship between plasma levels of IAIPs and endothelial cell activation in septic patients imply that IAIPs ameliorate endothelial inflammation (33). These *in vitro* and *in vivo* findings could in part explain the some of the beneficial properties, by which IAIPs attenuate the effects of sepsis *in vivo*. Furthermore, it was reported that the decrease in plasma IAIPs corresponded to the severity of Dengue virus infection and that the recovery from Dengue fever symptoms paralleled with the restoration of plasma IAIPs (34). Consequently, HRG and IAIPs could potentially represent two endogenous proteins that play key complementary overlapping roles to preserve intravascular blood cell and vascular endothelial cellular homeostasis in sepsis related disorders (**Figure 1**) (26, 32).

## High Mobility Group Box-1 as an Important Damp

High mobility group box-1 (HMGB1), a highly conserved nonhistone nuclear protein, plays a particularly important role as proinflammatory factor in the extracellular space through the stimulation of plural receptors: receptor for advanced glycation end products, toll-like receptor-4/2 (35). HMGB1 is released from not only necrotic cells but also many kinds of living cells under different conditions of stress including hypoxia, ischemia, and stimulation by cytokines (36, 37). The neutralization of extracellular HMGB1 by specific monoclonal antibody (mAb) was reported to inhibit influenza virus (H1N1)-induced pneumonia and improve the survival of infected mice (38, 39). This antibody effect was additive to that obtained by the antiviral drug, peramivir (38). Therefore, the above-mentioned HRG regulation of HMGB1 translocation and HMGB1-induced cytokine production in vascular endothelial cells suggest that HRG (23) as well as anti-HMGB1 mAb (38, 39) could contribute to the maintenance of vascular endothelial cellular homeostasis through the control of HMGB1 resulting in inhibition of inflammation induced by influenza infection. Moreover, it was suggested that HMGB1 may facilitate NETs and NETosis (40). Based on these findings, it is speculated that HMGB1 may play a crucial role in the development of lung inflammation in COVID-19 (16, 41). In fact, Chen et al. (42) determined the plasma levels of HMGB1 in severe COVID-19 patients and found a significant elevation compared with those of healthy volunteers.

## Conclusion

These results from models of animal sepsis and the clinical findings suggest that decreases in HRG and IAIPs concentrations could represent excellent biomarkers to evaluate the severity of sepsis mainly caused by bacterial infections (25, 30). At present, we have no data on plasma levels of HRG or IAIPs in patients with COVID-19. However, it remains plausible that there might be a common process present in the cascade of ARDS/MOF irrespective of the etiology of sepsis, bacterial or viral. In addition to ARDS, venous thromboembolism or endovasculitis may occur in COVID-19 because recent analysis warned of the high incidence of these comorbidities in COVID-19 infections (3, 4). The findings of the protection of vascular endothelial cells by HRG (23) as well as IAIPs (33), and the prevention of platelet aggregation by IAIPs (31) and erythrocyte aggregation by HRG (43) support the functional role of these plasma proteins against venous thromboembolism. Therefore, considerable attention should be paid to the dynamic changes in the plasma levels of HRG and IAIPs, especially with regard to severe cases of COVID-19, in order to delineate further the pathogenesis of this disorder.

## Author Contributions

All authors contributed to the article and approved the submitted version.

## Funding

This work was supported in part by grants from the Japan Agency of Medical Research and Development (AMED, JP19 im0210109) and from a Grant-in-Aid for Scientific Research (no.19H03408) to MN and from National Institutes of Health (NIH) 1R21NS095130, 1R21NS096525, 2R01HD057100, and 2R44 NS084575 to BS. The content is solely the responsibility of the authors and does not necessarily represent the official views of the National Institutes of Health.

## Conflict of Interest

The authors declare that the research was conducted in the absence of any commercial or financial relationships that could be construed as a potential conflict of interest.

## Abbreviations

COVID-19, coronavirus disease 2019; HRG, histidine-rich glycoprotein; IAIPs, inter α-inhibitor proteins; MOF, multiple organ failure; NETs, neutrophil extracellular traps; SARS-CoV-2, severe acute respiratory syndrome coronavirus-2.

## References

[1] Wiersinga WJ Rhodes A Cheng AC Peacock S Prescott HC. Pathophysiology, transmission, diagnosis, and treatment of coronavirus disease 2019 (COVID-19). JAMA (2020) 324:782–93. 10.1001/jama.2020.12839 32648899

[2] Liao D Zhou F Luo L Wang H Xia J Gao Y. Haematological characteristics and risk factors in the classification and prognosis evaluation of COVID-19: a retrospective cohort study. Lancet (2020) 7:e671–8. 10.1016/S2352-3026(20)30217-9 PMC7351397 32659214

[3] Ackermann M Verleden SE Kuehnei M Haverich A Welte T Laenger F. Pulmonary vascular endothelialitis, thrombosis, and angiogenesis in COVID-19. New. Engl J Med (2020) 383:120–8. 10.1056/NEJMoa2015432 PMC7412750 32437596

[4] Goshua G Pine AB Meizlish ML Chang C-H Zhanf H Bahel P. Endotheliopathy in COVID-19-associated coagulopathy: evidence from a single-centre, cross-sectional study. Lancet Haematol (2020) 7:e575–82. 10.1016/S2352-3026(20)30216-7 PMC7326446 32619411

[5] Wichmann D Sperhake JP Lütgehetmann M Steurer S Edler C Heinemann A. Autopsy Findings and Venous Thromboembolism in Patients With COVID-19: A Prospective Cohort Study. Ann Intern Med (2020) 173:268–77. 10.7326/M20-2003 PMC7240772 32374815

[6] Mahmud E Dauerman HL Welt FGP Messenger JC Rao SV Grines C. Management of Acute Myocardial Infarction During the COVID-19 Pandemic: A Position Statement From the Society for Cardiovascular Angiography and Interventions (SCAI), the American College of Cardiology (ACC), and the American College of Emergency Physicians (ACEP). J Am Coll Cardiol (2020) 76:1375–84. 10.1016/j.jacc.2020.04.039 PMC7173829 32330544

[7] Avula A Nalleballe K Narula N Sapozhnikov S Dandu V Toom S. COVID-19 presenting as stroke. Brain Behav Immun (2020) 87:115–9. 10.1016/j.bbi.2020.04.077 PMC7187846 32360439

[8] Brinkmann V Reichard U Goosmann C Fauler B Uhlemann Y Weiss DS. Neutrophil extracellular traps kill bacteria. Science (2004) 303:1532–5. 10.1126/science.1092385 15001782

[9] Papanyannopoulos V Metzler KD Hakkim A Zychlinsky A. Neutrophil elastase and myeloperoxidase regulate the formation of neutrophil extracellular traps. J Cell Biol (2010) 191:677–91. 10.1083/jcb.201006052 PMC3003309 20974816

[10] Leppkes M Knopf J Naschberger E Lindemann A Singh J Herrmann I. Vascular occlusion by neutrophil extracellular traps in COVID-19. EBioMedicine (2020) 58:102925. 10.1016/j.ebiom.2020.102925 32745993 PMC7397705

[11] Middleton EA He XY Denorme F Campbell RA Ng D Salvatore SP. Neutrophil extracellular traps contribute to immunothrombosis in COVID-19 acute respiratory distress syndrome. Blood (2020) 136:1169–79. 10.1182/blood.2020007008 PMC7472714 32597954

[12] Kimball AS Obi AT Diaz JA Henke PK. The Emerging Role of NETs in Venous Thrombosis and Immunothrombosis. Front Immunol (2016) 7:236. 10.3389/fimmu.2016.00236 27446071 PMC4921471

[13] Radermecker C Detrembleur N Guiot J Cavalier E Henket M d’Emal C. Neutrophil extracellular traps infiltrate the lung airway, interstitial, and vascular compartments in severe COVID-19. J Exp Med (2020) 217:e20201012. 10.1084/jem.20201012 32926097 PMC7488867

[14] Schurink B Roos E Radonic T Barbe E Bouman CSC de Boer HH. Viral presence and immunopathology in patients with lethal COVID-19: a prospective autopsy cohort study. Lancet Microbe (2020) 1:e290–9. 10.1016/S2666-5247(20)30144-0 PMC7518879 33015653

[15] Barnes BJ Adrover JM Baxter-Stoltzfus A Borczuk A Cools-Lartigue J Crawford JM. Targeting potential drivers of COVID-19: Neutrophil extracellular traps. J Exp Med (2020) 217:e20200652. 10.1084/jem.20200652 32302401 PMC7161085

[16] Cicco S Cicco G Racanelli V Vacca A. Neutrophil Extracellular Traps (NETs) and Damage-Associated Molecular Patterns (DAMPs): Two Potential Targets for COVID-19 Treatment. Med Inflamm (2020) 2020:7527953. 10.1155/2020/7527953 PMC7366221 32724296

[17] Poon IK Patel KK Davis DS Parish CR Hulett MD. Histidine-rich glycoprotein: the Swiss Army knife of mammalian plasma. Blood (2011) 117:2093–101. 10.1182/blood-2010-09-303842 20971949

[18] Wake H Mori S Liu K Morioka Y Teshigawara K Sakaguchi M. Histidine-rich glycoprotein prevents septic lethality through regulation of immunothrombosis and inflammation. EBioMedicine (2016) 9:180–94. 10.1016/j.ebiom.2016.06.003 PMC4972547 27333033

[19] Kuroda K Wake H Mori S Hinotsu S Nishibori M Morimatsu H. Decrease in histidine-rich glycoprotein as a novel biomarker to predict sepsis among systemic inflammatory response syndrome. Crit Care Med (2018) 46:570–6. 10.1097/CCM.0000000000002947 29303798

[20] Yoshii Y Wake H Nishimura Y Teshigawara K Wang D Nishibori M. An Evaluation of the Activity of Histidine-Rich Glycoprotein on Differentiated Neutrophil-Like Cells from Human Cell Lines. J Pharmacol Exp Ther (2020) 375:406–13. 10.1124/jpet.120.000182 33077479

[21] Takahashi Y Wake H Sakaguchi M Yoshii Y Teshigawara K Wang D. Histidine-rich glycoprotein stimulates human neutrophil phagocytosis and prolongs survival through CLEC1A. J Immunol (2021) 206(4):737–50. 10.4049/jimmunol.2000817 PMC7851742 33452125

[22] Gao S Wake H Gao Y Wang D Mori S Liu K. Histidine-rich glycoprotein ameliorates endothelial barrier dysfunction through regulation of NF-κB and MAPK signal pathway. Br J Pharmacol (2019) 176:2808–24. 10.1111/bph.14711 PMC6609555 31093964

[23] Gao S Wake H Sakaguchi M Wang D Takahashi Y Teshigawara K. Histidine-rich glycoprotein inhibits high-mobility group box-1 –mediated pathways in vascular endothelial cells through Clec-1A. iScience (2020) 23:101180. 10.1016/j.isci.2020.101180 32498020 PMC7267745

[24] MacQuarrie JL Stafford AR Yau JW Leslie BA Vu TT Fredenburgh JC. Histidine-rich glycoprotein binds factor XIIa with high affinity and inhibits contact-initiated coagulation. Blood (2010) 117:4134–41. 10.1182/blood-2010-07-290551 21304106

[25] Nishibori M Wake H Morimatsu H. Histidine-rich glycoprotein as an excellent biomarker for sepsis and beyond. Crit Care (2018) 22:209. 10.1186/s13054-018-2127-5 30119699 PMC6097411

[26] Lord MS Melrose J Day AJ Whitelock JM. The inter-a-trypsin inhibitor family: versatile molecules in biology and pathology. J Histochem Cytochem (2020) 68:907–27. 10.1369/0022155420940067 PMC7711241 32639183

[27] Singh K Zhang LX Bendelja K Heath R Murphy S Sharma S. Inter-alpha inhibitor protein administration improves survival from neonatal sepsis in mice. Pediatr Res (2010) 68:242–7. 10.1203/PDR.0b013e3181e9fdf0 PMC2928396 20520583

[28] Wu R Cui X Lim YP Bendelja K Zhou M Simms HH. Delayed administration of human inter-α inhibitor proteins reduces mortality in sepsis. Crit Care Med (2004) 32:1747–52. 10.1097/01.ccm.0000132903.14121.0e 15286553

[29] Baek YW Brokat S Padbury JF Pinar H Hixson DC Lim YP. Inter-alpha inhibitor proteins in infants and decreased levels in neonatal sepsis. J Pediatr (2003) 143:11–5. 10.1016/S0022-3476(03)00190-2 12915817

[30] Lim YP Bendelja K Opal SM Siryaporn E Hixson DC Palardy JE. Correlation between mortality and the levels of inter-alpha inhibitors in the plasma of patients with severe sepsis. J Infect Dis (2003) 188:919–26. 10.1086/377642 12964125

[31] Chaaban H Keshari RS Silasi-Mansat R Popescu NI Mehta-D’Souza P Lim YP. Inter -alpha inhibitor protein and its associated glycosaminoglycans protect against histone-induced injury. Blood (2015) 125:2286–96. 10.1182/blood-2014-06-582759 PMC4383802 25631771

[32] Htwe SS Wake H Liu K Teshigawara K Stonestreet BS Lim YP. Inter-α inhibitor proteins maintain neutrophils in a resting state by regulating shape and reducing ROS production. Blood Adv (2018) 2:1923–34. 10.1182/bloodadvances.2018018986 PMC6093744 30093530

[33] Stober VP Lim YP Opal S Zhuo L Kimata K Garantziotis S. Inter-α inhibitor ameliorates endothelial inflammation in sepsis. Lung (2019) 197:361–9. 10.1007/s00408-019-00228-1 PMC6888858 31028466

[34] Koraka P Lim YP Shin MD Setiati TE Mairuhu AT van Gorp EC. Plasma levels of inter-alpha inhibitor proteins in children with acute Dengue virus infection. PloS One (2010) 5:e9967. 10.1371/journal.pone.0009967 20386596 PMC2850310

[35] Andersson U Tracey KJ. HMGB1 is a therapeutic target for sterile inflammation and infection. Ann Rev Immunol (2011) 29:139–62. 10.1146/annurev-immunol-030409-101323 PMC4536551 21219181

[36] Nishibori M Mori S Takahashi HK. Anti-HMGB1 monoclonal antibody therapy for a wide range of CNS and PNS diseases. J Pharmacol Sci (2019) 140:94–101. 10.1016/j.jphs.2019.04.006 31105025

[37] Nishibori M Wang D Ousaka D Wake H. High mobility group box-1 and blood-brain barrier disruption. Cells (2020) 9:2650. 10.3390/cells9122650 PMC7764171 33321691

[38] Hatayama K Nosaka N Yamada M Yashiro M Fujii Y Tsukahara H. Combined effect of anti-high-mobility grou box-1 monoclonal antibody and peramivir against influenza A virus-induced pneumonia in mice. J Med Virol (2019) 91:361–9. 10.1002/jmv.25330 30281823

[39] Nosaka N Yashiro M Yamada M Fujii Y Tsukahara H Liu K. Anti-high mobility group box-1 monoclonal antibody treatment provides protection against influenza A virus (H1N1)-induced pneumonia in mice. Crit Care (2015) 19:249. 10.1186/s13054-015-0983-9 26067826 PMC4490661

[40] Wang Y Du F Hawez A Morgelin M Thorlacius H. Neutrophil extracellular trap-microparticle complexes trigger neutrophil recruitement via high-mobility group box 1 (HMGB1)-toll-like receptors (TLR2)/TLR4 signalling. Br J Pharmacol (2019) 176:3350–63. 10.1111/bph.14765 PMC6692579 31206609

[41] Yang H Wang H Andersson U. Targeting inflammation driven by HMGB1. Front Immunol (2020) 11:484. 10.3389/fimmu.2020.00484 32265930 PMC7099994

[42] Chen L Long X Xu Q Tan J Wang G Cao Y. Elevated serum levels of S100A8/A9 and HMGB1 at hospital admission are correlated with inferior clinical outcomes in COVID-19 patients. Cell Mol Immunol (2020) 17:992–4. 10.1038/s41423-020-0492-x PMC7332851 32620787

[43] Zhong H Wake H Liu K Gao Y Teshigawara K Sakaguchi M. Effects of Histidine-rich glycoprotein on erythrocyte aggregation and hemolysis: Implications for a role under septic conditions. J Pharmacol Sci (2018) 136:97–106. 10.1016/j.jphs.2017.11.003 29544683

